# Inhibition of NLRP3 enhances pro-apoptotic effects of FLT3 inhibition in AML

**DOI:** 10.1186/s12964-025-02046-w

**Published:** 2025-01-28

**Authors:** Helene Sieberer, Michela Luciano, Diana Amend, Constantin Blöchl, Anna Eglseer, Alina Steinkellner, Sebastian Rieser, Ancuela Andosch, Philip Steiner, Laura Hummer, Peter W. Krenn, Hieu-Hoa Dang, Christian G. Huber, Fritz Aberger, Theresa Neuper, Jutta Horejs-Hoeck

**Affiliations:** 1 Department of Biosciences and Medical Biology, Paris-Lodron University Salzburg, Hellbrunner Strasse 34, Salzburg, 5020 Austria; 2 Center for Tumor Biology and Immunology, Paris-Lodron University Salzburg, Salzburg, 5020 Austria; 3 Cancer Cluster Salzburg, Salzburg, 5020 Austria; 4 Institute of Pharmacology, Medical Faculty, Johannes Kepler University Linz, Linz, 4020 Austria

## Abstract

FLT3 mutations occur in approximately 25% of all acute myeloid leukemia (AML) patients. While several FLT3 inhibitors have received FDA approval, their use is currently limited to combination therapies with chemotherapy, as resistance occurs, and efficacy decreases when the inhibitors are used alone. Given the highly heterogeneous nature of AML, there is an urgent need for novel targeted therapies that address the disease from multiple angles. Recent research has identified the NLRP3 inflammasome as a potential new driver in AML. Here, we investigated the efficacy of different NLRP3 inhibitors in targeting AML cells in vitro. Our findings reveal that NLRP3 inhibition induces cell cycle arrest as well as signs of senescence in multiple AML cell lines. In contrast, NLRP3 inhibition selectively induced apoptosis in FLT3 mutant AML cell lines, but not in FLT3 wild-type AML cells. Moreover, we show that NLRP3 inhibition impairs FLT3 signaling by reducing both FLT3 expression as well as downstream signaling in FLT3 mutant cells. A database analysis revealed a strong positive correlation between FLT3 and NLRP3 in cancer, which was particularly evident in AML patients. Strikingly, the simultaneous inhibition of NLRP3 and FLT3 markedly enhanced apoptosis in FLT3-ITD mutant AML cells, but not in FLT3 wild-type cells. In summary, this study reveals a promising combined therapeutic strategy specifically targeting NLRP3/FLT3-ITD positive AML blasts in vitro, highlighting a potential new avenue for AML treatment.

**Supplementary Information:**

The online version contains supplementary material available at 10.1186/s12964-025-02046-w.

## Introduction

Acute myeloid leukemia (AML) is a hematopoietic malignancy that originates from abnormal proliferation and impaired differentiation of myeloid cells in the bone marrow and peripheral blood. It is the most prevalent form of acute leukemia in adults with low complete remission rates [1]. The age and fitness of patients as well as specific mutations and cytogenetic abnormalities strongly influence the disease outcome [2]. For example, mutations in the Fms-like tyrosine kinase 3 (FLT3) gene are associated with reduced complete remission and increased relapse rates, resulting in poor prognosis for patients [3]. *FLT3* mutations include internal tandem duplications (ITD) in 20% of all AML patients and mutations in the tyrosine kinase domain (TKD) in another 5–10% [4–6]. These genetic alterations result in constitutive and FLT3 ligand (FLT3-L)-independent receptor activation [7] that promotes aberrant upregulation of the PI3K-AKT, RAS-MAPK and STAT5 pathways [8] involved in cell proliferation and survival [9]. Even though different FLT3 tyrosine kinase inhibitors (TKIs) have recently been approved by the FDA (Midostaurin, Gilterinib, and Quizartinib), these agents are only employed in combination with the cytostatics Cytarabine and Daunorubicin due to their insufficient efficacy as a monotherapy [10–14]. The lack of monotherapeutic efficacy, refractory disease, acquired resistance, and relapse following FLT3 TKI treatment underscores the need to exploit novel drugs for effective combinatory therapy for AML patients [11, 15, 16].

The NLRP3 inflammasome has been recently identified as a novel driver in AML [17–21]. This protein complex formed by NLRP3, ASC (adapter protein), and pro-caspase-1 is an important component of the innate immune system [22, 23]. After inflammasome oligomerization, pro-caspase-1 is activated and triggers the cleavage of the cytokine precursors pro-interleukin (IL)-1β and pro-IL-18 and the pore-forming protein gasdermin D into their mature forms, leading to pyroptotic cell death [24]. Carey et al. reported that IL-1β promotes the growth of myeloid progenitor cells, contributing to an inflammatory environment and AML progression [25]. Additionally, oncogenic *KRAS* mutations were identified to induce NLRP3 inflammasome activation, promoting myeloproliferation [17]. Furthermore, Zhong et al. demonstrated that upregulation of NLRP3 promotes AML progression and mortality in mice, identifying NLRP3 as a potential driver of AML [18]. We recently confirmed these studies by highlighting that deletion and pharmacological inhibition of NLRP3 induce apoptosis in MOLM-13 AML cells [21], further underscoring the important role of NLRP3 in AML. Here, we extended our previous studies by showing that the combination of NLRP3 and low-dose FLT3 inhibitors strongly boosts apoptosis specifically in FLT3-ITD mutant AML cells.

## Results

### NLRP3 inhibition induces cell cycle arrest and signs of senescence

**Fig. 1:**
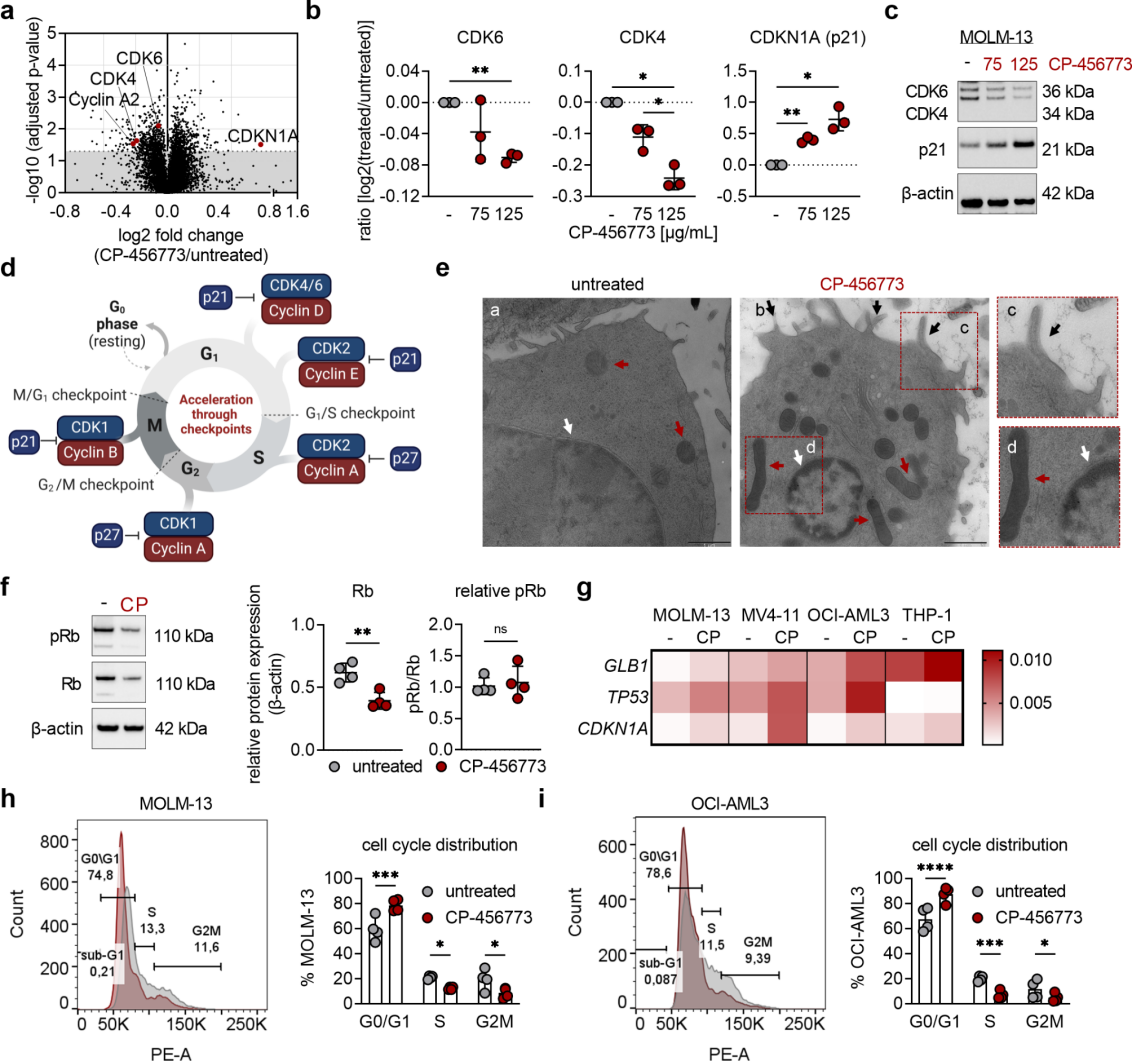
NLRP3 inhibition induces cell cycle arrest in AML cells. **a** MOLM-13 cells (untreated (-), 75 and 125 µg/mL CP-456773) were incubated for 24 h before being processed for HPLC-MS based proteomics analysis allowing relative quantification of around 7000 proteins throughout these three conditions. Volcano plot illustrating the differences (log2FC) in protein expression between untreated and CP-456773-treated (125 µg/mL) MOLM-13 cells. Adjusted *p*-values were obtained from a paired t-test applying Benjamini-Hochberg correction. Gray background: not significantly regulated, white background: significantly regulated proteins between the two groups (adjusted *p*-value of 0.05) with a false discovery rate (FDR) of 10%. **b** Protein expression values of CDK6, CDK4 and CDKN1A (p21) obtained by described proteomics analysis (three biological replicates). **c** MOLM-13 cells (untreated (-), 75 and 125 µg/mL CP-456773) were incubated for 24 h before being processed for Western blot analysis of CDK6, CDK4, p21 (β-actin serving as a loading control). One representative immunoblot out of three is shown. **d** Graphic illustration of the eukaryotic cell cycle (adapted after [65], Created in BioRender. Sieberer, H. (2025) https://BioRender.com/z97n519). **e** Transmission electron micrographs of untreated (panel a) and CP-456773-treated MOLM-13 cells (125 µg/mL, panel b) cultured for 48 h. Scale bars correspond to 1 μm. In panel b, black arrows = irregularity of the plasma membrane (= panel c); red arrows = mitochondrial elongation and hyperfusion (= panel d), and white arrow = pyknosis (= panel d). **f** Untreated (-) and 125 µg/mL CP-456773 treated (CP) MOLM-13 cells were incubated for 48 h before being processed for Western blot analysis of pRb and Rb total (β-actin serving as a loading control). One representative immunoblot out of four is shown. Rb was quantified relative to β-actin and pRb was normalized to the total Rb levels. **g** Heatmap showing the mean relative mRNA expression of *GLB1*, *TP53* and *CDKN1A* to the house keeping gene *RPLP0* of untreated and 125 µg/mL CP-456773 treated MOLM-13, MV4-11, OCI-AML3 and THP-1 cells after 48 h incubation time (*n* = 6). **h + i** Cell cycle analysis of untreated and 125 µg/mL CP-456773-treated MOLM-13 (**h**) and OCI-AML3 (**i**) cells after 24 h of incubation employing propidium iodide staining (*n* = 4). Dots in graphs indicate individual replicates; bars represent mean ± SD. A paired t-test was used for the comparison between two groups (**f**), a one-way ANOVA with Tukeys’ post-hoc test (**b**) and a two-way ANOVA with Šídák’s post-hoc test (**h**, **i**) was performed for multiple comparisons. Significance levels are defined as follows: *, *p* ≤ 0.05; **, *p* ≤ 0.01; ***, *p* ≤ 0.001; ****, *p* ≤ 0.0001

As we described previously, pharmacological inhibition or genetic knockout of NLRP3 reduces cell viability in MOLM-13 AML cells by inducing apoptosis [21]. To gain further insights into the mechanisms by which NLRP3 disruption exerts its growth-inhibitory effects in AML cells, we performed whole-cell proteomics of MOLM-13 cells treated with the NLRP3 inhibitor CP-456773. This analysis revealed differential expression of cell cycle regulators, as the protein expression of the cyclin-dependent kinases 4 and 6 (CDK4/6) was significantly downregulated, whereas the protein level of their natural inhibitor p21 (CDKN1A) was elevated upon NLRP3 inhibition (Fig. 1a-b). These findings were confirmed by Western blot analysis in the NLRP3-expressing AML cell lines [21] MOLM-13 (Fig. 1c, Suppl. Figure 1a), MV4-11, OCI-AML3 and with less pronounced effects in THP-1 cells (Suppl. Figure 1b). CDK4/6 promote the transition from the G1 phase to the S phase, whereas p21 inhibits their activity and thus halts the cell cycle (Fig. 1d) [26]. Given the observed downregulation of CDK4/6, we hypothesized there would be cell cycle arrest in AML cells after NLRP3 inhibition. Transmission electron microscopy (TEM) indeed revealed signs of senescence and cell cycle arrest, including irregular plasma membranes, mitochondrial hyperfusion and structured heterochromatin at the nuclear periphery in NLRP3-inhibited MOLM-13 cells (Fig. 1e, panel b-d). In line with this, we observed signs of senescence including reduced expression of Retinoblastoma protein (Rb) in MOLM-13 cells (Fig. 1f) and upregulation of *GLB1*, *TP53* and *CDKN1A* in all tested AML cell lines after NLRP3 inhibition (Fig. 1g). Furthermore, we detected an increased percentage of MOLM-13, OCI-AML3 (Fig. 1h-i), MV4-11 and THP-1 cells (Suppl. Figure 1c-d) in the G0/G1 phase upon NLRP3 inhibition. This set of data shows that NLRP3 inhibition induces cell cycle arrest in AML cells.

### NLRP3 inhibition induces apoptosis and downregulation of the FLT3 pathway

**Fig. 2:**
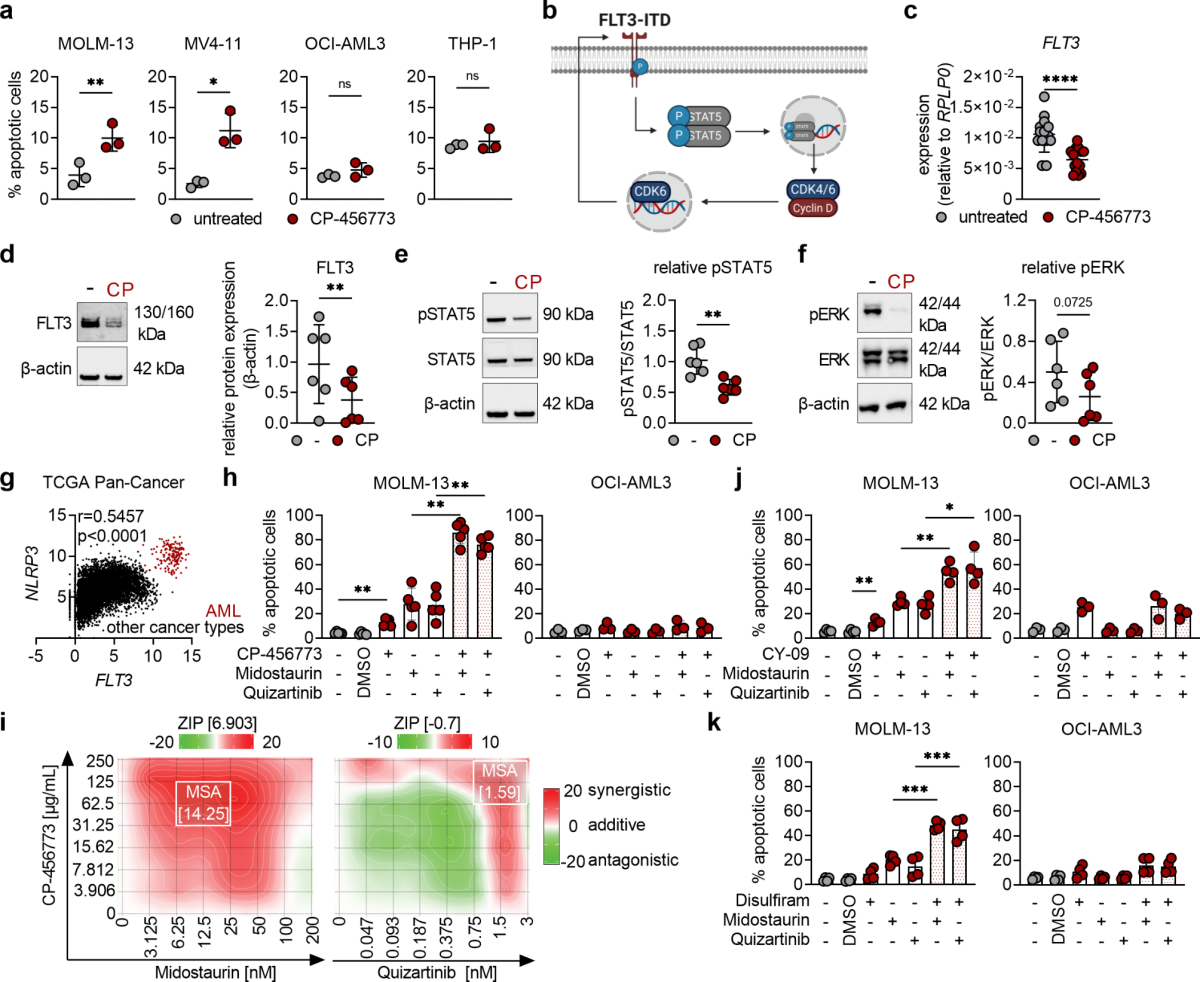
Combined inhibition of NLRP3 and FLT3 strongly enhance apoptosis in FLT3-ITD mutant AML cells. **a** Bar chart showing the percentage of apoptotic (sum of Annexin V^+^/7-AAD^−^ and Annexin V^+^/7-AAD^+^) MOLM-13, MV4-11, OCI-AML3 and THP-1 cells that were either left untreated or treated with 125 µg/mL CP-456773 48 h after seeding (*n* = 3). **b** Graphic illustration of the FLT3 pathway in AML cells (Created in BioRender. Sieberer, H. (2025) https://BioRender.com/g03k424). **c** *FLT3* mRNA levels were determined by qRT-PCR (*n* = 13, relative mRNA expression to the housekeeping gene *RPLP0*) in untreated and CP-456773-treated (125 µg/mL) MOLM-13 cells after 48 h incubation. **d/e/f** MOLM-13 cells were either left untreated (-) or treated with 125 µg/mL CP-456773 (CP) and incubated for 48 h before being processed for Western blot analysis of FLT3 (**d**), phosphorylated STAT5 (Tyr694), total STAT5 (**e**), phosphorylated ERK1/2 and total ERK1/2 (**f**) (β-actin serving as a loading control). One representative immunoblot out of six is shown. Densiometric quantification of the Western blots: FLT3 expression was compared to the house keeping β-actin (**d**), phosphorylated proteins were compared to their total counterparts (**e/f**). **g** Correlation of FLT3 and NLRP3 expression using the TCGA Pan-Cancer dataset via UCSC Xena [30]. **h** Bar charts showing the percentage of apoptotic MOLM-13 and OCI-AML3 cells 48 h after treatment with 125 µg/mL CP-456773, 50 nM Midostaurin, 0.75 nM Quizartinib or a combination of CP-456773 with either Midostaurin or Quizartinib (*n* = 5 for MOLM-13, *n* = 3 for OCI-AML3). 0.1% DMSO was used as a solvent control. **i** Dose-response synergy analysis of CP-456773 with Midostaurin or Quizartinib in MOLM-13 cells after 48 h treatment using SynergyFinder [31]. The most synergistic area (MSA) is highlighted (white rectangle) with the respective MSA score. The mean viability values of three independent experiments were used for the generation of the shown heatmap. **j/k** Bar charts showing the percentage of apoptotic MOLM-13 and OCI-AML3 cells 48 h after treatment with 10 µM CY-09 (**j**) or 75 nM Disulfiram (**k**), 50 nM Midostaurin, 0.75 nM Quizartinib or a combination of NLRP3 and FLT3 inhibitors (*n* = 4 for MOLM-13, *n* = 3–4 OCI-AML3). 0.1% DMSO was used as a solvent control. For statistics, a paired t-test was used for the analysis between two groups (**a**, **c**-**f**) and a one-way ANOVA with Šídák’s post-hoc test was used for multiple comparisons (**h**, **j**, **k**). Significance levels are defined as follows: *, *p* ≤ 0.05; **, *p* ≤ 0.01; ***, *p* ≤ 0.001 ****, *p* ≤ 0.0001; ns, not significant

We next tested the potency of CP-456773 to induce apoptosis in different AML cell lines. Surprisingly, although all tested cell lines showed cell cycle arrest (Fig. 1h + i, Suppl. Figure 1c + d), only MOLM-13 and MV4-11 cells (FLT3-ITD mutant) showed significantly more apoptotic cell death, while OCI-AML3 and THP-1 cells (FLT3-wild-type (wt)) did not show any significant increase in apoptosis upon NLRP3 inhibition (Fig. 2a). Given that NLRP3 inhibition significantly increases apoptosis in FLT3-ITD mutant cells, but not in FLT3-wt or healthy peripheral blood mononuclear cells (PBMC’s) (Fig. 2a, Suppl. Figure 2a), we speculated that the induced apoptosis might be dependent on the FLT3 pathway. In this context, it is noteworthy that NLRP3 inhibition resulted in downregulation of CDK6 (Fig. 1a-c), as Uras et al. demonstrated that CDK6 serves as a transcriptional regulator of the *FLT3* gene in AML cells [27]. Furthermore, it is well known that constitutively active FLT3 phosphorylates and activates STAT5 [28]. This can further promote CDK6 expression [29], thereby providing positive feedback in the FLT3 pathway (illustrated in Fig. 2b). In accordance, we found that NLRP3 inhibition led to a notable reduction in both FLT3 transcription and protein levels in FLT3-ITD mutant but not in FLT3-wt AML cells (Fig. 2c + d, Suppl. Figure 2b). In addition, NLRP3 inhibition markedly reduced the phosphorylation of the FLT3 downstream effectors STAT5 and ERK (Fig. 2e + f), indicating decreased activity of these transcription factors. Thus, these data suggest that NLRP3 inhibition correlates with a decrease in FLT3 signaling.

### Combined inhibition of NLRP3 and FLT3 strongly enhances apoptosis in AML cells

Based on the findings that NLRP3 inhibition impairs FLT3 signaling, we hypothesized that the combined inhibition of NLRP3 and FLT3 might be of particular interest in AML therapy. Thus, we first analyzed the co-expression profiles of FLT3 and NLRP3 using the TCGA Pan-Cancer data via UCSC Xena [30]. Independent of the cancer type, there is a significant positive correlation between FLT3 and NLRP3 expression. Strikingly, the co-expression of NLRP3 and FLT3 is exceptionally pronounced in AML, exceeding the levels observed in all other investigated cancer types (Fig. 2g). Therefore, we hypothesized that NLRP3 inhibition-mediated downregulation of FLT3 might increase the susceptibility of AML cells to FLT3 inhibition. To test this hypothesis, we utilized nanomolar to picomolar concentrations of the FDA-approved FLT3 inhibitors Midostaurin and Quizartinib, which induced low levels of apoptosis in MOLM-13 cells when administered alone, while having no effects on apoptosis in THP-1 cells (Suppl. Figure 2c). Strikingly, our findings revealed that the combined inhibition of NLRP3 and FLT3 remarkedly enhanced apoptosis in FLT3-ITD mutant AML cell lines but not in FLT3-wt AML cells or in healthy PBMC’s (Fig. 2h, Suppl. Figure 2d). Quantitative analysis using the web tool SynergyFinder [31] revealed that the combination of CP-456773 and Midostaurin acts synergistically in FLT3-mutant MOLM-13 cells (Fig. 2i). Our findings were further substantiated by testing two additional NLRP3 inhibitors (CY-09 and Disulfiram), which significantly enhanced apoptosis in MOLM-13 cells but not in OCI-AML3 cells when used in combination with FLT3 inhibitors (Fig. 2j, k). In conclusion, these experiments demonstrate that the combined inhibition of NLRP3 and FLT3 significantly increases apoptosis specifically in FLT3-ITD/NLRP3^+^ AML cells as compared to the single treatments. This further underlines the potential relevance and importance of simultaneously targeting NLRP3 and FLT3 in AML.

## Discussion

Acute myeloid leukemia is a highly aggressive hematopoietic disease with a 5-year survival rate of only 13.6% [32]. Both large chromosomal rearrangements and genetic mutations play an important role in the onset and progression of the disease [1]. However, none of the identified genetic aberrations are sufficient to induce AML when occurring alone. It is only the combination of matching initiating and cooperating mutations that contributes to the clonal expansion of the affected myeloid cell, thereby promoting disease evolution [33]. AML treatment remains a significant challenge, as each AML genome contains at least one nonsynonymous mutation relevant for pathogenesis in addition to other recurrent mutated genes [34].

The standard treatment for AML has not significantly changed in the past four decades. The “7 + 3 regime” involves the intravenous administration of the chemotherapeutic agent Daunorubicin for three days, including Cytarabine for seven days [35, 36]. Especially frail elderly patients have a low likelihood of complete remission, as they do not tolerate intense chemotherapy well [37]. Consequently, older or unfit patients are treated with the hypomethylating agent Azacitidine in combination with the BCL-2 inhibitor Venetoclax. This treatment is better tolerated and results in a similar overall survival as intense chemotherapy [38, 39]. Nevertheless, complete remission and survival rates remain low, underscoring the necessity for new targeted therapies in AML [40].

In the past few years, different FLT3 tyrosine kinase inhibitors (TKIs) have been approved by the FDA for the treatment of AML. However, these TKIs are applied only in combination with Cytarabine and Daunorubicin due to their insufficient monotherapeutic efficacy [10–14]. Moreover, upon long-term exposure to a TKI, AML blasts develop resistance against the inhibitor, which compromises TKI efficacy [41]. In addition, chemotherapy can result in elevated FLT3 ligand levels in the serum of patients, which can also reduce the effectiveness of FLT3 inhibitors [11, 16]. Therefore, potential avenues for future therapies may include the development of new combinations of FLT3 TKI with other inhibitors. In this context, Yang et al. demonstrated that the combination of low-dose CDK4/6 and FLT3 inhibitors helped to overcome TKI resistance and successfully reduced AML cell growth in vitro [42]. Moreover, the combination of Venetoclax and Quizartinib was shown to significantly improve survival rates of immunodeficient NSG mice engrafted with MV4-11 or MOLM-13 cells, as compared to untreated mice [43]. Similar effects were observed upon simultaneous inhibition of FLT3-ITD and menin-MLL, targeting two key drivers in AML, in NSG mice engrafted with MV4-11 cells [44]. These reports highlight the potential success of combination therapy for AML patients.

In this regard, we identified NLRP3 as a novel target for potential combination therapy in AML as we show that NLRP3 inhibition renders AML cells sensitive to low doses of FLT3 inhibitors. While recent studies have demonstrated that NLRP3 acts as a potential driver of AML [17–21], the exact mechanisms by which NLRP3 contributes to leukemogenesis in AML remain to be elucidated. Here we show that inhibition of NLRP3 induces G1 cell cycle arrest accompanied by upregulation of p21 and downregulation of CDK4/6. This is consistent with the study by Nasrollahzadeh et al., which showed that the FDA-approved NLRP3 inhibitor Disulfiram [45] induces G1 cell cycle arrest in MCF-7 cells in line with reduced CDK4/6 and increased p21 expression [46]. Furthermore, we found that NLRP3 inhibition reduces FLT3 expression, and we hypothesize that this is due to the downregulation of CDK6, which has been identified as a transcription factor for FLT3 [27]. Interestingly, the combination of NLRP3 inhibitors including CP-456773, CY-09 and Disulfiram with low concentrations of Midostaurin and Quizartinib specifically and significantly enhanced apoptosis in FLT3-ITD AML cell lines, leaving FLT3-wt cells unaffected. In addition, co-expression analysis using the TCGA Pan-Cancer dataset via UCSC Xena [30] revealed a positive correlation between NLRP3 and FLT3 in more than 30 different cancer types. Co-expression of both NLRP3 and FLT3 was particularly pronounced in AML patients, emphasizing the importance of targeting both, NLRP3 and FLT3, in AML. It was previously shown that high expression of pro-inflammatory genes in AML patients is associated with poor response to FLT3 inhibitors [47, 48]. In line with this, inhibition of inflammation, e.g.: treatment with IRAK1/4 inhibitors or glucocorticoids, was demonstrated to enhance cell death induced by FLT3 inhibitors [49, 50]. Inhibition of NLRP3 may exert a similar effect by mitigating inflammatory responses, which could render FLT3-ITD mutant AML cells more vulnerable to FLT3 inhibitors, thereby enhancing pro-apoptotic effects.

A limitation of this study is the use of a high concentration of the NLRP3 inhibitor CP-456773. Of note, clinical trials with CP-456773/MCC950 were prematurely stopped due to unexpected liver toxicity [51]. Nevertheless, CP-456773/MCC950 remains a prevalent NLRP3 inhibitor in both in vitro and in vivo studies of numerous diseases, including diabetes, atherosclerosis, myocardial infarction, neurodegenerative diseases, and myeloid malignancies [17, 52–57]. To strengthen the main message of this study, we also employed CY-09 and Disulfiram, both of which are NLRP3 inhibitors. The results obtained with CP-456773 (Fig. 2h) were validated, as the combination treatments with CY-09 or Disulfiram and FLT3 inhibitors (Fig. 2j + k) produced similarly strong pro-apoptotic effects in FLT3-ITD mutant AML cell lines, despite being used at significantly lower concentrations. Further research, including in vivo studies, will help to comprehensively elucidate the precise molecular interplay between NLRP3 and FLT3, in order to strengthen the finding that combined inhibition of NLRP3 and FLT3 could represent a new therapeutic strategy for AML patients.

## Materials and methods

### AML cell lines, and culture conditions

This study was conducted in accordance with the approved guidelines of the World Medical Association’s Declaration of Helsinki and the guidelines of the Ethics Committee of the Province of Salzburg. The AML cell lines MOLM-13, MV4-11 (both FLT3-ITD mutant), OCI-AML3 and THP-1 (both FLT3-wt; all purchased from Leibniz-Institut DSMZ GmbH) were cultured in RPMI-1640 medium (Sigma-Aldrich, Catalog number: R0883) supplemented with 10% heat-inactivated fetal bovine serum (FBS; Catus Biotech, Catalog number: BS-2020-500), 1% penicillin and streptomycin (Sigma-Aldrich, Catalog number: P4333), and 2 mM L-glutamine (Sigma-Aldrich, Catalog number: G7513). PBMC’s were collected from fresh buffy coats of healthy donors via gradient density centrifugation using Histopaque^®^-1077 (Sigma-Aldrich, Catalog Number: 10771). ACK lysis buffer was used to remove red blood cells. PBMC’s were cultured in complete RPMI-1640 medium as described above. All cells were cultured at 37 °C, 5% CO_2_ in a humified atmosphere and regularly screened for mycoplasma contamination (MycoAlert™ PLUS Mycoplasma Detection Kit; Lonza, Catalog Number: LT07-705) following the manufacturer’s instructions.

2 × 10^5^ cells/mL were seeded in appropriate cell culture plates and incubated for the indicated time points. CP-456773 (Sigma-Aldrich, Catalog number: PZ0280) was dissolved in sterile water, CY-09 (Tocris, Catalog number: 6436), Disulfiram (Selleckchem, Catalog number: S1680), Midostaurin (Sigma-Aldrich, Catalog number: M1323), Quizartinib (Selleckchem, Catalog number: S1526) and Bortezomib (MedChemExpress, Catalog number: HY-10227) were dissolved in DMSO.

### Quantitative real time polymerase chain reaction (qRT-PCR)

Cell pellets of cultured cells were directly lysed in Tri Reagent^®^ (Sigma-Aldrich, Catalog number: T9424) and RNA was isolated according to the manufacturer’s instructions. Briefly, chloroform was added, and the lysates were spun down. LPA (Sigma-Aldrich, Catalog number: 56575) was used as a carrier and mixed with the RNA-containing phase. Isopropanol was used to precipitate the RNA, and thereafter 70% ethanol was added for washing. RevertAid H Minus M-MulV reverse transcriptase (Thermo Fisher Scientific, Catalog number: EP0451) was used to generate complementary DNA (cDNA) in an iCycler Thermal Cycler (Bio-Rad). To determine gene expression levels by qRT-PCR, a Luna^®^ Universal Probe qPCR Master Mix (New England BioLabs^®^ Inc, Catalog number: M3003) was used and the amplification reactions were performed on a Rotorgene 3000 (Qiagen Instruments). To determine relative gene/mRNA expression, the large ribosomal protein P0 (*RPLP0*) was used as a reference gene. Relative mRNA expression (x) was calculated as x = 2^−Δct^, where Δct represents the difference between the threshold cycle (ct) of the target gene and the reference gene. Following primers (Sigma-Aldrich) were used: *FLT3*: forward 5’-ACCTCAAGTGCTCGCAGAAGCA-3’, reverse 5’-GTTAGCCTTTCTATTCCAGACTCC-3’; *GLB1*: forward 5’-CACTCCACAATCAAGACCGAAGC-3’, reverse 5’-CTGTGCTGCATAGGGTGAGTTG-3’; *TP53*: forward 5’-CCTCAGCATCTTATCCGAGTGG-3’, reverse 5’-TGGATGGTGGTACAGTCAGAGC-3’; *CDKN1A*: forward 5’-AGGTGGACCTGGAGACTCTCAG-3’, reverse 5’-TCCTCTTGGAGAAGATCAGCCG-3’; *RPLP0*: forward 5’-GGCACCATTGAAATCCTGAGTGATGTG-3’, reverse 5’-TTGCGGACACCCTCCAGGAAG-3’.

### Western blot

Cell pellets were directly lysed in 80 µL of 2x Laemmli sample buffer (Bio-Rad, Catalog number: 1610737) supplemented with 5% β-mercaptoethanol (Sigma-Aldrich, Catalog number: M6250). The lysates were applied and separated on 4–12% NuPAGE Bis-Tris gradient gels (Life Technologies, Catalog number: NP0321) and then blotted onto a 0.45 μm nitrocellulose membrane (Bio-Rad, Catalog number: 1620115) using a semi-dry transfer system. 5% nonfat dry milk in 1x TBS containing 0.1% Tween 20 was used to block unspecific binding sites on the membrane. All antibodies were purchased from Cell Signaling and used according to the manufacturer’s instructions: CDK6 (3136), CDK4 (12790), p21 (2947), phosphorylated Rb (Ser780, 8190), total Rb (9309), FLT3 (3462), phosphorylated STAT5 (Tyr694, 9351), total STAT5 (9363), phosphorylated p44/42 MAPK (= ERK1/2, Thr202/Tyr204, 9106), total p44/42 (9102), β-actin (4970), HRP-linked anti-rabbit secondary antibody (7074) and HRP-linked anti-mouse secondary antibody (7076). A ChemiDox™ MP Imaging System was used for chemiluminescent detection (Thermo Fisher, Catalog number: 34580) of proteins. ImageJ software (NIH) was used for densiometric quantification of Western blots. Phosphorylated proteins were normalized to the respective total protein bands and total proteins were quantified relative to the loading control β-actin.

### Flow cytometric analyses

The AML cell lines were seeded in 24- or 48-well plates at a density of 2 × 10^5^ cells/mL and harvested according to manufacturer’s instructions for the chosen readout. Flow cytometric data were detected with a BD FACS Canto II and analyzed using FlowJo (FlowJo v10.7.1, BD Biosciences).

For cell cycle analysis, cells were seeded, treated as indicated and harvested after 24 and 48 h of incubation. The cells were stained with FxCycle™ PI/RNase Staining Solution according to the manufacturer´s instructions (Invitrogen, Catalog number: F10797). Viable cells were identified using the fixable viability dye eFluor™ 780 (Invitrogen, Catalog number: 65-0865-18).

Apoptosis of inhibitor treated cells was measured after 48 h of incubation using an Annexin V Apoptosis Detection Kit according to the manufacturer´s instructions (Invitrogen, Catalog number: 88-8006-74). Apoptotic cells are presented as the sum of the percentage of Annexin V^+^/7-AAD^−^ and Annexin V^+^/7-AAD^+^ cells.

### Synergy assay

2 × 10^5^ cells/mL (2 × 10^4^ cells/well) were seeded in flat bottom 96 well plates (Greiner, Catalog number: 655180) and treated with serial twofold dilutions of the drugs of interest. Untreated cells served as a positive control (= 100% viability) and cells treated with 10 µM Bortezomib (MedChemExpress, Catalog number: HY-10227) were used as a negative control (= 0% viability). 48 h post treatment, cell viability was measured using the CellTiter-Blue Cell Viability Assay (Promega, Catalog number: G8081) on the Infinite 200 PRO reader (Tecan) according to the manufacturer’s instructions. The data was normalized according to the positive and negative controls. The mean of three independent experiments was determined and the expected drug combination responses thereof were calculated based on the ZIP reference model using SynergyFinder. The interaction between two drugs can be defined as follows: a ZIP value of less than − 10 indicates antagonistic effects, a value between − 10 and + 10 indicates an additive effect, and a value greater than + 10 indicates synergistic effects [31].

### Co-expression analysis of FLT3 and NLRP3 using the UCSC Xena browser

The data provided by the UCSC Xena browser (https://xenabrowser.net/) was used for the co-expression analysis of NLRP3 and FLT3 in more than 30 different cancer types using the TCGA Pan-Cancer dataset. In total, 12.839 samples were used for the analysis including 173 AML samples [30].

### Proteomics

#### Chemicals

Dithiothreitol (DTT, ≥ 99.5%), formic acid (FA, 98.0-100%), iodoacetamide (IAA, ≥ 99.0%), sodium dodecyl sulfate (SDS, ≥ 99.5%) and triethylammonium bicarbonate (TEAB, pH 8.5, 1 mol/L) were obtained from Sigma-Aldrich (Vienna, Austria). Acetonitrile (ACN, ≥ 99.9%) and methanol (MeOH, ≥ 99.9%) were obtained from VWR International (Vienna, Austria). Ammonia (25%) and ortho-phosphoric acid (85%) were purchased from Merck (Burlington, MA, USA). Trypsin (sequencing grade modified, porcine) was obtained from Promega (Madison, WI, USA). A MilliQ Integral 3 instrument (Millipore, Billerica, MA, USA) was used for deionization of water.

#### Cell culture

MOLM-13 cells were seeded in a 48-well plate at a density of 2 × 10^5^/mL (1 × 10^5^/500µL/well). Cells were treated with 75 and 125 µg/mL of CP-456,773 and incubated for 24 h at 37 °C and 5% CO_2_. Biological replicates were generated by conducting this treatment scheme on three consecutive days. Cells were washed thoroughly with PBS before conducting proteomics sample preparation.

#### Sample preparation

S-Trap mini columns (Protifi, Huntington, NY, USA) were employed according to the manufacturer´s instructions with minor adjustments: a cell pellet of approximately 1 × 10^6^ cells was lysed in 5% SDS and 50 mmol/L TEAB (pH 7.55) at 95 °C for 5 min followed by sonication in a Bioruptor device (Diagenode, Liège, Belgium) for 10 min. After a centrifugation step, protein content was analyzed by a Pierce BCA Protein assay kit (Thermo Fisher Scientific, Vienna, Austria). Denaturation and reduction of proteins were performed by supplementation of DTT to 40 mmol/L and incubation at 95 °C for 10 min. Reduced cysteine residues were alkylated by the addition of IAA to a concentration 80 mmol/L and incubation at 21 °C in the dark for 30 min. After a precipitation step and intense washing, 10 µg of trypsin were solubilized in 50 mmol/L TEAB (pH 8.5), added to the S-Trap matrix and incubated at 37 °C for 18 h. Peptides were eluted and subsequently dried at 30 °C using a vacuum centrifuge. Samples were resuspended in 100 mmol/L TEAB (pH 8.5) to a concentration of 1.0 mg/mL. 100 µg of peptides of each sample were labeled by a TMT 10plex™ kit (Thermo Fisher Scientific) according to the manufacturer´s instructions. Samples were labeled with the tags 126 to 130 C. All nine samples were pooled and dried at 30 °C in a vacuum centrifuge. The combined sample was resuspended in H_2_O + 20 mmol/L ammonium formate (pH 10.0) to a concentration of 1.8 mg/mL.

#### High-performance liquid chromatography and mass spectrometry

The pooled peptide sample was subjected to high-pH reversed phase fractionation without any prior purification. This off-line fractionation was carried out on an Agilent 1100 Series capillary LC system (Santa Clara, CA, USA). The peptides were separated on two sequentially linked Gemini NX-C18 columns (150 × 2.0 mm i.d., 3 μm particle diameter, 110 Å pore size) purchased from Phenomenex Inc. (Aschaffenburg, Germany) that were connected by a short 20 μm i.d. connective tubing. Mobile phases A (H_2_O + 20 mmol/L ammonium formate, pH10) and B ((90.0% ACN / 10.0% H_2_O) + 20 mmol/L ammonium formate, pH 10) were prepared according to Dwivedi et al. and Gilar et al. [58, 59]. A stepped linear gradient was applied: 1.0% B for 15.0 min, 1.0 − 30.0% B for 185.0 min, 30.0 − 60.0% for 30.0 min, 80.0% B for 30.0 min and 1.0% B for 40.0 min. The flow rate was set to 150 µL/min, the column oven temperature to 40 °C and 100.0 µl of sample were injected. 30 independent fractions were collected by an integrated automatic sample fraction collector system at uniform time slices starting at time point 12.0 min until time point 264.0 min. These 30 fractions were pooled into six fractions employing sample concatenation as reviewed by Yang et al. increasing orthogonality of the two chromatographic dimensions both relying on reversed phase separation principles [60]. Pooled fractions were dried at 45 °C in a vacuum centrifuge and upon dryness resuspended in 16 µl of H_2_O + 0.1% FA.

As a second dimension, acidic reversed phase HPLC was employed using a 2000 mm µPAC™ C18 column (PharmaFluidics, Ghent, Belgium). These nano scale chromatographic separations were carried out on a nanoHPLC instrument (UltiMate™ U3000 RSLCnano, Thermo Fisher Scientific, Germering, Germany) at a flow rate of 300 nL/min and a column oven temperature of 50 °C. Mobile phase solution A contained H_2_O + 0.10% FA and mobile phase B ACN + 0.10% FA. After solvent B was kept at 1.0% B for 5.0 min, a linear gradient to 40.0% B in 595.0 min was applied. This gradient was followed by a purging step at 90.0% B for 30.0 min. The column was re-equilibrated at 1.0% B for 100.0 min. 1.0 µl of each fraction was injected using a microliter pick-up mode (5.0 µl loop volume). Each fraction was measured once.

The nanoHPLC was hyphenated to a quadrupole-Orbitrap hybrid mass spectrometer (Thermo Scientific QExactive Plus benchtop quadrupole-Orbitrap mass spectrometer) via a Nanospray Flex ion source (both from Thermo Fisher Scientific, Bremen, Germany). The source was equipped with a SilicaTip emitter with 360 μm o.d., 20 μm i.d. and a tip i.d. of 10 μm purchased from New Objective (Woburn, MA, USA). The mass spectrometer was operated with following instrument settings: spray voltage of 1.5 kV, S-lens RF level of 55.0, capillary temperature of 320 °C and an MS1 AGC target of 3e6 in an m/z range of 400–2000 with a maximum injection time of 100 ms. A MS1 scan at a resolution setting of 70,000 at 200 m/z was followed by 15 data-dependent MS2 scans at a resolution of 35,000 at m/z 200. Target peptides were fragmented by HCD at 32.0 NCE in a 2.0 m/z isolation window with an AGC target of 1e5 and a maximum injection time of 100 ms. A dynamic exclusion setting of 30 s was applied. Pierce LTQ Velos ESI Positive Ion Calibration Solution from Life Technologies (Vienna, Austria) was used for calibration of the instrument.

#### Data evaluation

Acquired raw data was evaluated using MaxQuant software (v1.6.3.4) [61] in default settings correcting for isotope impurities in TMT reagents (provided by the manufacturer). Uniprot database entries including both Swiss-Prot as well as TrEMBL entries for homo sapiens (access: 10.03.2019) were provided for MaxQuant protein identification [62]. Obtained protein groups were further processed using Perseus (v1.6.6.1): Protein groups were filtered removing potential contaminants, proteins that were only identified by site and reverse sequence matches [63]. Only those protein groups providing quantitative values for all nine reporter ion channels were further processed, including log2-transformation and normalization by subtraction of the median. For statistical analysis and data representation, the R software version 3.6.1 as well as GraphPad Prism version 8.0.2. (GraphPad Software, San Diego, CA, USA) were employed [64].

### Transmission electron microscopic (TEM) preparation and visualization

MOLM-13 cells were either left untreated or treated with 125 µg/mL of CP-456773 for 48 h and then processed for TEM imaging as described previously [21]. The cells were high-pressure frozen (HPF), cryosubstituted and then embedded in epoxy resin. Thereafter, the samples were trimmed and cut to ultrathin sections (~ 70 nm). Samples on Formvar-coated copper grids were then transferred to the transmission electron microscope (TEM) and images were recorded on a LEO 912 AB Omega TEM at 80 kV. Digital images were recorded with a bottom-mounted 2 K CCD camera from Tröndle TRS Sharp Eye (Tröndle, Moorenweis, Germany). All TEM images were filtered at zero energy loss. iTEM 5.0 software (Olympus SIS, Münster, Germany) was used for TEM implementation and the recording process.

### Statistical analysis

Statistical analyses were performed with GraphPad Prism 9 software (GraphPad Software, San Diego, CA, USA). Data was tested for normality and appropriate statistical tests were used: Statistical analyses were performed by a paired t-test for the analysis between two groups, one-way ANOVA with Tukey’s or Šídák’s post-hoc test or a two-way ANOVA with Šídák’s post hoc test for multiple comparisons. Significance levels are defined as follows: *, *p* ≤ 0.05; **, *p* ≤ 0.01; ***, *p* ≤ 0.001; ****, *p* ≤ 0.0001; ns, not significant.

## Electronic supplementary material

Below is the link to the electronic supplementary material.

Supplementary Material 1

## Data Availability

The mass spectrometry proteomics data have been deposited in the ProteomeXchange Consortium (https://www.ebi.ac.uk/pride/archive) via the PRIDE partner repository with the dataset identifier PXD026293.

## List of Abbreviations

AML: Acute myeloid leukemia
ASC: Apoptosis-associated speck-like protein containing a caspase recruitment domain
BM: Bone marrow
Caspase 1: Cysteine-aspartic acid protease
CDK4/6: Cyclin dependent kinase 4/6
CDKN1A/p21: Cyclin-dependent kinase inhibitor 1 A
ERK / p44/42: Mitogen-activated protein kinase
FDA: U.S. food and drug administration
FLT3: Fms like tyrosine kinase receptor 3
HPLC: High-performance liquid chromatography
IL-18: Interleukin 18
IL-1b: Interleukin 1-beta
ITD: Internal tandem duplications
MS: Mass spectrometry
NLRP3: Nucleotide-binding oligomerization domain, leucine rich repeat and pyrin domain containing protein 3
PBMC: Peripheral blood mononuclear cells
Rb: Retinoblastoma protein
STAT5: Signal transducers and activators of transcription 5
TEM: Transmission electron microscopy
TKD: Tyrosine kinase domain
TP53: Tumor suppressor protein 53
