# Supplementary material for: Inhibition of NLRP3 enhances pro-apoptotic effects of FLT3 inhibition in AML
Source: Cell Commun Signal. 2025 Jan 28;23:53. doi: 10.1186/s12964-025-02046-w (PMC11773904; doi:10.1186/s12964-025-02046-w)
Supplement: Supplementary file 1 — Supplementary Material 1 [file 12964_2025_2046_MOESM1_ESM.docx]

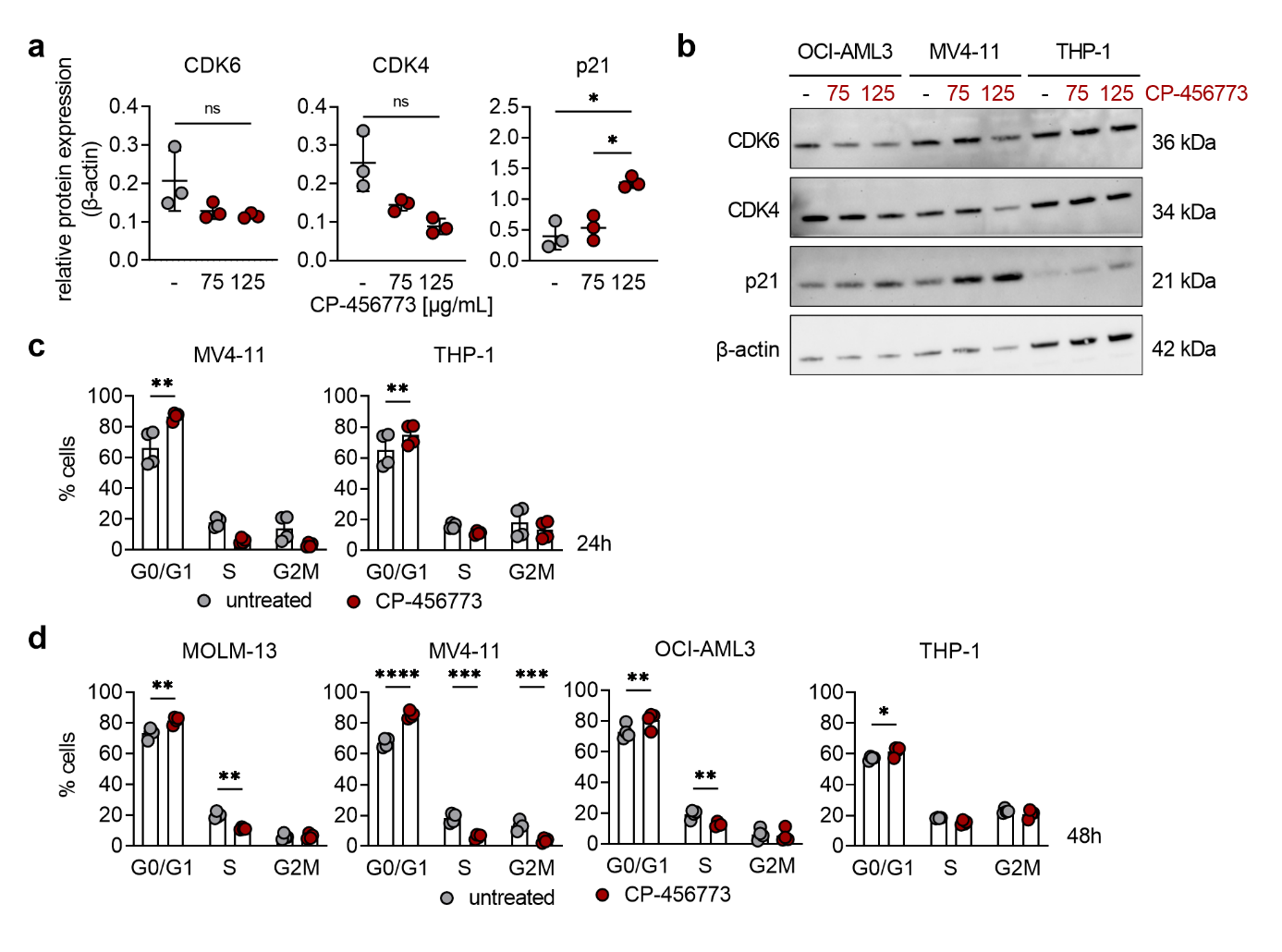
**Supplementary Figure 1: NLRP3 inhibition induces cell cycle arrest in AML cells. a** Densiometric quantification of CDK6, CDK4 and p21 of Western blots shown in Figure 1c using β-actin as a loading control. **b** OCI-AML3, MV4-11 and THP-1 cells (untreated (-), 75 and 125 µg/mL CP-456773) were incubated for 24 h before being processed for Western blot analysis of CDK6, CDK4, p21 (β-actin serving as a loading control). One representative immunoblot out of three is shown. **c/d** Cell cycle analysis of untreated and 125 µg/mL CP-456773-treated AML cells after 24 h (c) and 48 h (d) of incubation, respectively (n=4). A two-way ANOVA with Šídák's post-hoc test (c+d) was performed for multiple comparisons. Significance levels are defined as follows: *, p ≤ 0.05; **, p ≤ 0.01; ***, p ≤ 0.001; ****, p ≤ 0.0001.


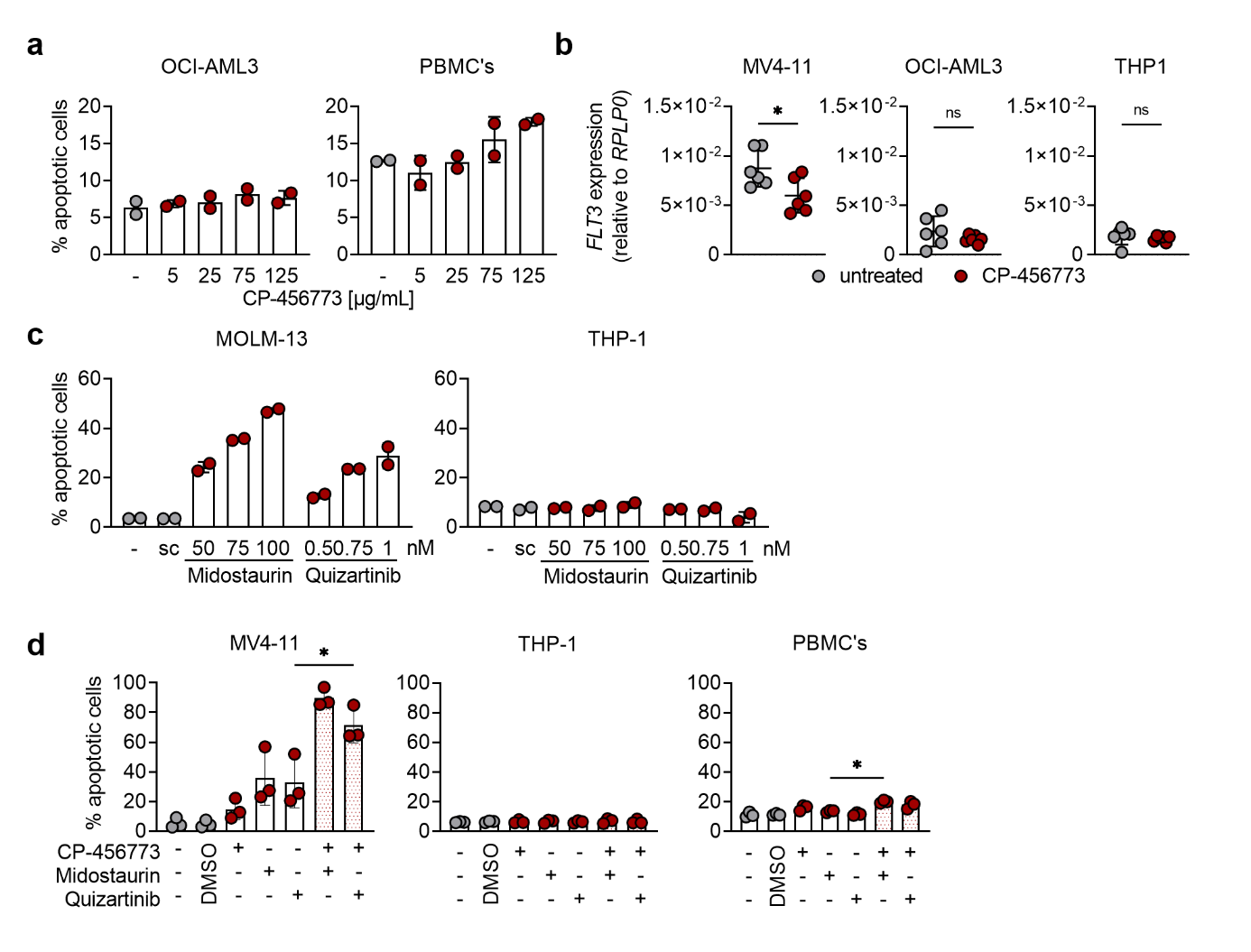
**Supplementary Figure 2: NLRP3 inhibition reduces FLT3 expression and boosts pro-apoptotic effects of FLT3 inhibitors in FLT3-mutant but not in FLT3-wt AML cells. a** Bar chart showing the percentage of apoptotic (sum of Annexin V+/7-AAD- and Annexin V+/7-AAD+) OCI-AML3 and freshly isolated peripheral blood mononuclear cells (PBMC’s) that were treated with increasing concentrations of CP-456773 48 h after seeding (n=2). **b** *FLT3* mRNA levels were determined by qRT-PCR (n=6, relative mRNA expression to the housekeeping gene *RPLP0*) in untreated and CP-456773-treated (125 µg/mL) MV4-11, OCI-AML3 and THP-1 cells after 48 h of incubation. **c** Bar charts showing the percentage of apoptotic MOLM-13 and THP-1 cells 48 h after seeding (sc = 0.1 % DMSO used as a solvent control, n=2). **d** Bar charts showing the percentage of apoptotic MV4-11, THP-1 cells and PBMC’s 48 h after treatment with 125 µg/mL CP-456773, 50 nM Midostaurin, 0.75 nM Quizartinib or a combination thereof. 0.1 % DMSO was used as a solvent control (n=3). For statistics, a paired t-test was used for the analysis between two groups (b) and a one-way Anova with Šídák's (d) post-hoc test was used for multiple comparisons. Significance levels are defined as follows: *, p ≤ 0.05; ns, not significant.
